# Development of tilt-scan system for differential phase contrast scanning transmission electron microscopy

**DOI:** 10.1093/jmicro/dfac002

**Published:** 2022-01-15

**Authors:** Yuji Kohno, Akiho Nakamura, Shigeyuki Morishita, Naoya Shibata

**Affiliations:** EM Research and Development Department 2, JEOL Ltd., 3-1-2, Musashino, Akishima, Tokyo 196-8558, Japan; EM Research and Development Department 2, JEOL Ltd., 3-1-2, Musashino, Akishima, Tokyo 196-8558, Japan; EM Research and Development Department 2, JEOL Ltd., 3-1-2, Musashino, Akishima, Tokyo 196-8558, Japan; Institute of Engineering Innovation, School of Engineering, The University of Tokyo, Yayoi 2-11-16, Bunkyo-ku, Tokyo 113-8656, Japan; Nanostructures Research Laboratory, Japan Fine Ceramic Center, 2-4-1 Mutsuno, Atsuta-ku, Nagoya 456-8587, Japan

**Keywords:** DPC STEM, diffraction contrast, beam scan system

## Abstract

Differential phase contrast (DPC) scanning transmission electron microscopy can directly visualize electromagnetic fields inside a specimen. However, their image contrast is not only sensitive to the electromagnetic fields in the sample, but also the changes in diffraction conditions such as sample bends or thickness changes. These additional contrasts are called diffraction contrasts, and sometimes make it difficult to extract pure electromagnetic field information from the experimental DPC images. In this study, we developed a beam scan system that can acquire many DPC images from the same sample region with arbitrarily varying incident beam tilt angles to the sample. Then, these images are precisely averaged to form tilt-scan averaged DPC images. It is shown that the diffraction contrast can be effectively reduced in the tilt-scan averaged DPC images.

## Introduction

Differential phase contrast scanning transmission electron microscopy (DPC STEM) visualizes electric and/or magnetic field distributions in a specimen by measuring the changes in the convergent-bean electron diffraction (CBED) pattern induced by the specimen electromagnetic fields using segmented or pixelated detectors placed on the detector plane [1]. The incident electron beam is deflected by the electromagnetic fields in the specimen through the Coulomb and/or Lorentz force. By assuming weak phase object approximation, the field magnitude is proportional to the shift of the bright-field disk’s center of mass on the detector plane [1–3]. However, the CBED pattern is also strongly affected by the changes in diffraction conditions due to local strain, specimen bend, thickness gradient, chemical inhomogeneities, etc. Thus, the DPC image contrasts are sensitive to the local electromagnetic field in the specimen and the local changes in diffraction conditions. This additional contrast is often called diffraction contrast [4,5]. Thus, it is difficult to estimate the electromagnetic field distribution by DPC STEM quantitatively if strong diffraction contrast is present in the image.

**Fig. 1.**
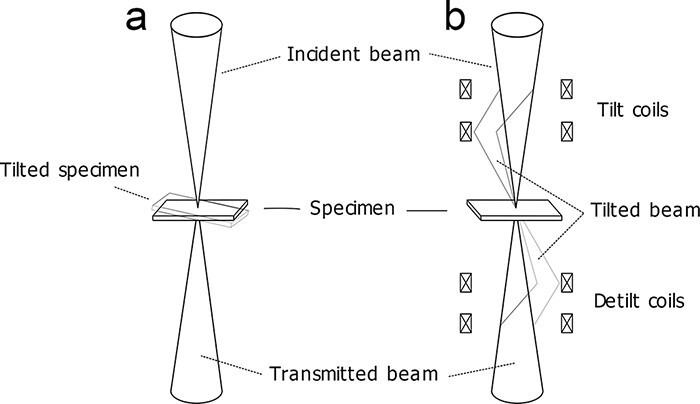
Schematic illustrations of STEM methods showing how to tilt the incident beam angles to the sample. (a) The sample is mechanically tilted by the sample stage. (b) The beam is tilted by a set of deflectors, while the BF disk position on the detector plane is fixed by another set of deflectors for DPC imaging.List: STEM, scanning transmission electron microscopy; BF, bright field; DPC, differential phase contrast.

It has been reported that the diffraction contrast in DPC images can be effectively reduced by averaging multiple DPC images with different sample tilt conditions [4,5]. This method is schematically illustrated in Fig. 1a. This averaging method is deemed effective because the diffraction contrast is more sensitive to small changes in the relative incident angle than the target electromagnetic field. After averaging a series of tilted DPC images, the diffraction contrast can be averaged out, and the electromagnetic field component remains in the image contrast. However, in the case of practical experiments, particular care must be paid to not degrade the image quality of the averaged DPC images. For example, the optical parameter deviations originated from the change in defocus, and the drift of aberrations or the sample shift caused by mechanical tilt should be precisely corrected and aligned during each image acquisition and post-image processing. Another problem is that these corrections and post-image processing are time-consuming, and require careful manual operations by users.

To improve the efficiency of such experiments, beam tilt in scanning probe may have more advantages as schematically illustrated in Fig. 1b. Moreover, since the reproducibility of the beam deflection system is much better than that of the mechanical sample tilt, it is promising to realize high-speed DPC image acquisition under various beam tilt conditions and for automatic image integration and averaging. In this case, it will be indispensable to minimize beam shift and geometrical aberrations induced by the beam tilt to avoid image degradation.

In the present study, we developed a new beam scan system for STEM that can arbitrarily control an incident beam’s tilt angle against a sample. Using this scan system, we can precisely average many DPC images at different tilt angles from the same sample region to form tilt-scan averaged DPC images without time-consuming post-image averaging processes. Furthermore, it will be shown that the diffraction contrast can be effectively reduced using this new scan/tilt control system.

## Methods

**Fig. 2.**
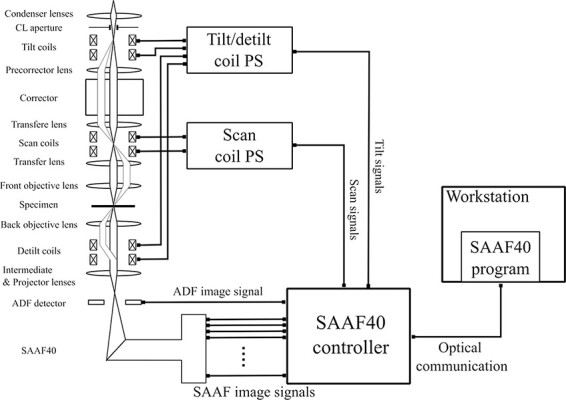
Schematic diagram of the tilt scan DPC STEM system. The tilt/detilt coils and their control systems are added to an aberration-corrected STEM.List:DPC, differential phase contrast; STEM, scanning transmission electron microscopy.

We have developed a tilt-scan system as schematically shown in Fig. 2. This system can automatically acquire and average multiple DPC images with varying beam tilt conditions. The base electron microscope is a JEM-ARM200F equipped with a magnetic field-free objective lens and a DELTA corrector [6] as a probe corrector, which realizes atomic-resolution STEM observation in a magnetic-field-free environment [7]. The conventional scan coils and power supply are configured to scan the electron probe across a sample without changing the incident beam tilt angle against the sample. In this study, we added a set of deflection coils (denoted as tilt coils) in between the aberration corrector and the illumination diaphragm (CL aperture) to control the incident electron beam angle to the sample. In addition, another set of deflection coils (denoted as detilt coils) is added between the objective and the intermediate lens to cancel the beam tilt induced by the tilt coils. The tilt/detilt-scan power supply synchronously controls the currents of the tilt and detilt coils, in order not to cause beam shifts on both specimen and detector planes during the beam tilt scanning. In these optical settings, the beam tilt on the sample plane does not induce a bright-field disk shift on the detector plane, avoiding any artefacts on DPC images due to the tilted beam scanning.

A segmented detector system, which we call SAAF40 system developed for this electron microscope [8], can simultaneously acquire annular dark field (ADF) and 40 segmented detection area signals in synchronization with the change of scan and tilt signals and form DPC images. The SAAF40 system, whose basic technique is previously reported, uses a scintillator, optical fiber bundles and photo-multiplier tubes (PMTs) for detecting scattered and/or transmitted electrons [9]. Using a scintillator and PMTs achieves a fast-sampling rate of up to 0.4 μs per pixel. Furthermore, the house-made operating software for the SAAF40 developed at the University of Tokyo can control scan and tilt-signal waveforms and sampling timing, which realizes flexible management of the scan and tilt–signal relationships.

## Results and discussion

In conventional high-resolution STEM, the deflection system for beam tilt needs to be placed above the objective lens, since there is not enough space between the objective lens and the specimen. When the beam on the sample is tilted by the deflection system above the objective lens, the beam does not pass through the center of the objective lens. Let ${C_S}$ be the spherical aberration coefficient, $\omega\ {\rm{and}}\ \bar \omega $ be the complex convergence angle and its complex conjugate. When the optic axis passes through the center of the objective lens, the geometrical aberration $u$ on the sample plane is

$$u = {C_S}{\omega ^2}\bar \omega .$$

When the beam is tilted on the sample plane by the complex angle $\alpha $, the geometrical aberration $u$ changes as follows.

$$u & = {C_S}{\left( {\omega + \alpha } \right)^2}\left( {\bar \omega + \bar \alpha } \right) = {C_S}{\alpha ^2}\bar \alpha + 2{C_S}\alpha \bar \alpha \omega + {C_S}{\alpha ^2}\bar \omega \cr & \quad+ \left( {2{C_S}\alpha \omega \bar \omega + {C_S}\bar \alpha {\omega ^2}} \right) + {C_S}{\omega ^2}\bar \omega $$

**Fig. 3.**
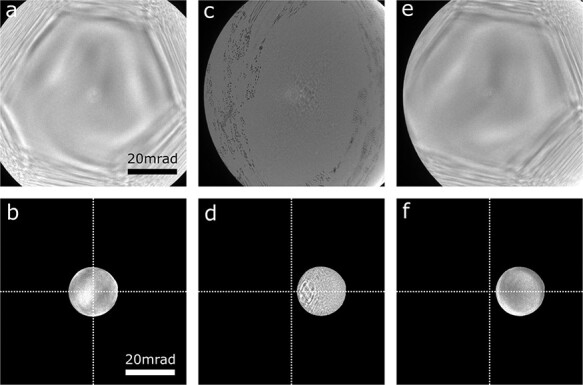
Ronchigram and CL aperture position with and without the beam tilt. (a) The Ronchigram without the beam tilt. The aberration-corrected area extends up to about 26 mrad. (b) The CL aperture position without the beam tilt. The CL aperture is aligned to the center of the aberration-corrected area. (c) The Ronchigram as a result of changing the beam tilt using CL alignment coil which is located near the scan coil. (d) The CL aperture position with the same condition as in (c). The beam is tilted about 12 mrad in the *x*-direction of this image. (e) The Ronchigram as a result of changing the beam tilt using the new tilt coil shown in Fig. 2. (f) The CL aperture position with the same condition as in (e). The beam is tilted to the same direction and amount as in (c) and (d).

The first to fourth terms on the right-hand side correspond to probe shift, defocus, astigmatism and coma aberration, respectively. Therefore, it is expected that the aberrations such as defocus, coma and astigmatism proportional to the spherical aberration of the objective lens may appear. Figures 3a and b show the Ronchigram and CL aperture position without the beam tilt. The geometrical aberration of the illumination system is mainly determined by the positive spherical aberration of the objective lens and the negative spherical aberration of the aberration corrector. The aberration-corrected area in the Ronchigram extends up to about 26 mrad by canceling these two aberrations. When the beam is tilted by the deflectors located between the objective lens and the aberration corrector (CL alignment coils), the center of the CL aperture and the negative spherical aberration of the corrector shift. For example, Fig. 3a and b change to Fig. 3c and d, respectively, after changing the beam tilt using the CL alignment coils. Here, the detilt coil is switched off so that the change of the beam tilt can be monitored on the detector plane. The change of the Ronchigram pattern is due to the misalignment between the center of the negative spherical aberration of the corrector and the positive spherical aberration of the objective lens. These aberrations severely degrade the image quality. Therefore, neither the CL alignment coils nor the scan coils located next to the CL alignment coils are suitable for tilting the beam for STEM.

As mentioned, we added tilt coils between the aberration corrector and the CL aperture. When the beam is tilted with these coils, Fig. 3a and b change to Fig. 3e and f. In this case, only the CL aperture position changes depending on the beam tilt angle. The Ronchigram pattern does not change because the center of the spherical aberration of the objective lens and the corrector do not change with beam tilting. The coma and astigmatism generated by the spherical aberration of the objective lens and the corrector are believed to be canceled. Thus, as long as the CL aperture stays in the aberration-corrected region, the image degradation due to the beam tilt should be suppressed. In the presence of off-axis aberrations which can occur due to transfer deviations between the corrector multipole and the front focal plane of the objective lens, the Ronchigram pattern may be changed if the beam is not only tilted but shifted by the tilt coils. The probe position accuracy on the sample plane during the beam tilt should be essential for DPC imaging and their image averaging. The probe position accuracy using the present beam tilting system will be described later.

In the new tilt-scan system, we also include averaging multiple images obtained with different beam tilt conditions. If the beam positions on the specimen are shifted depending on the tilt angles, the averaged image will be severely blurred. It is thus required to realize a ‘pure-tilt’ situation, which means just tilting the beam to the specimen while the beam position is fixed on the specimen. The ‘pure-tilt’ can be achieved for an ideal system by placing a single-stage deflector at an optically conjugate plane to the sample. However, it is challenging to realize ‘pure-tilt’ situations with a single-stage deflection system for two reasons in an actual system. One is the gaps in the deflection principal planes of the beam’s *x* and *y* projected trajectories due to an asymmetry of the tilt coils deflection field. The other is the gap at the position of the conjugate image planes to the sample plane of these trajectories due to the optical system aberration. Thus, the double-stage deflection system used in this study should be essential for practical systems.

There are four deflectors in the developed double stage deflection system for tilting the beam against the sample. Two of them are *x*- and *y*-direction deflectors of the first stage, and the other two are those of the second stage. The four deflection angles of these deflectors can be uniquely determined when the two-dimensional tilt angle is set to the target value at no shifts in the two-dimensional probe position on the specimen. Therefore, it is necessary to maintain this relationship to avoid image degradation during image acquisition. However, it is difficult to keep this relationship at high precision during the change of the beam tilt angle. This is because the waveform of the output deflection field is distorted due to the finite response time of the deflection system and is not proportional to the tilt-signal waveform. This distortion becomes remarkable for a tilt signal containing high-frequency components, such as a step function. One method to avoid this problem might be to use a single triangular function as the input signal like a precession method [10], which can minimize the waveform change. However, the precession method’s tilt angle control is not flexible and is only limited to circular or elliptical patterns.

**Fig. 4.**
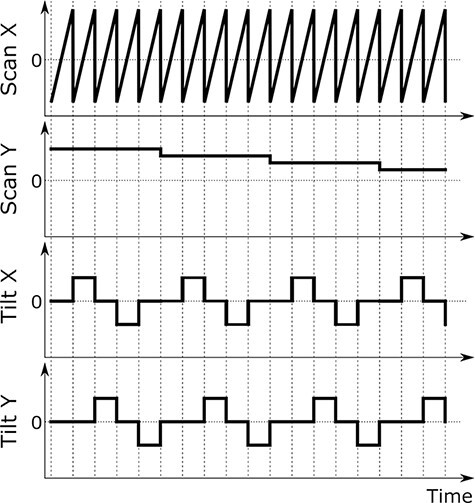
The schematic illustration of the scan and tilt waveforms. In this example, there are five tilt angles which are denoted as $\left( {{{\bf{\it{t}}}_{\bf{\it{x}}}},{\bf{\it{ }}}{{\bf{\it{t}}}_{\bf{\it{y}}}}} \right) = \left( {0,{\bf{\it{ }}}0} \right),{\bf{\it{ }}}\left( {{\bf{\it{T}}},{\bf{\it{ }}}0} \right),{\bf{\it{ }}}\left( {0,{\bf{\it{ T}}}} \right),{\bf{\it{ }}}\left( { - {\bf{\it{T}}},{\bf{\it{ }}}0} \right),{\bf{\it{ }}}\left( {0,{\bf{\it{ }}} - {\bf{\it{T}}}} \right)$. ${\bf{\it{T}}}$ is a constant. The scan X is repeated five times with different tilt conditions before the change in the scan Y signal. In this case, the same line signals with different tilt angles are continuously acquired.

In the new tilt-scan system, the main objective is to realize both pure and arbitrary tilt angle waveforms, similar to the sample-tilt method [4,5]. The tilt angle distribution can be flexibly selected and programmed by users. Here, the control of the scan and tilt signals can be synchronized to avoid acquiring STEM images during tilt angle changes. Figure 4 shows a schematic of the scan and tilt signals. A sawtooth waveform is used for the horizontal scan signal in a conventional STEM control system. Like the tilt waveform, the scan waveform also has a response time problem, and the distorted deflection field of the scan appears as image distortion. Therefore, there is a flyback time at the start of the horizontal scan to avoid such image distortion, where the image signals are not used and discarded. The changes in the tilt coil deflection fields can also be completed during this flyback time since the tilt coils and power supply can respond fast enough as the scanning system.

The image acquisition time increases in proportion to the number of tilt angles since the angle is changed only during the flyback time in this system. For example, the fastest acquisition time of 512 × 512 pixels image with 61 tilt angles becomes 36.6 s which is 61 times longer than the fastest acquisition time (0.6 s) of the image without beam tilt. Since a high-speed detection system using PMT is used, a practical image acquisition speed can be realized even with many tilt angles.

To minimize the influence of the sample drift, we repeatedly scan the beam at the same vertical sample position with different tilt angles and acquire image signals. In the example of Fig. 4, we set five different tilt angle conditions during the scan. As a result, the scan *y* signal is changed after the horizontal scan is repeated five times with five other tilt angle conditions.

In this system, the currents of four tilt and detilt coils are controlled as a linear combination of the tilt *x* and *y* signals. Thus, the system holds 16 proportional constants as control parameters for these coil currents, called deflection ratios. The eight deflection ratios of the tilt coils are determined as follows. Four of the eight ratios use fixed values calculated from the deflector placement. The other two are determined to minimize the shifts of the ADF images during the tilt *x* signal change. The other two are determined to reduce the shifts during the tilt *y* signal change. After calibrating the tilt deflection ratios, the detilt deflection ratios are adjusted similarly to the tilt deflection ratios. Thus, four of the eight deflection ratios use fixed values. The remaining four ratios are determined to minimize the beam shifts due to the tilt *x* and *y* signal changes at the detector plane observed using a charge-coupled device (CCD) camera.

**Fig. 5.**
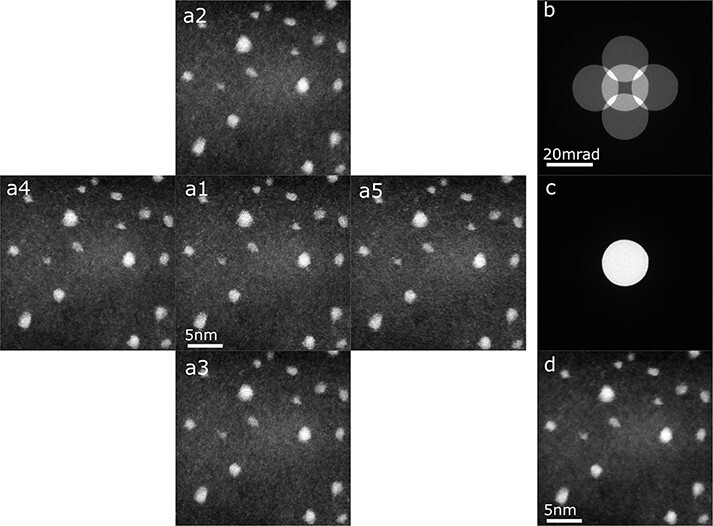
ADF STEM images of gold nanoparticles on carbon film under beam tilted conditions. The tilt angles have five values as schematically shown in Fig. 4. Here, the T value is 12 mrad. (a) Simultaneously acquired ADF STEM images with five different beam tilt angle conditions. (b) The BF disks on the detector plane with the detilt coils off. (c) The BF disks on the detector plane with the detilt coils on. (d) The average image of the five ADF STEM images with different tilt angles.List:ADF, annular dark field; STEM, scanning transmission electron microscopy, BF, bright field.

Figure 5a shows ADF STEM images of gold nanoparticles on carbon thin film acquired by the tilt-scan system under the five different beam tilt angle conditions. The pixel size of the images is 512 × 512, and the dwell time at each pixel is 38 μs/pixel. The tilt angles (in milliradians) of the center, right, top, left and bottom images are ${\left( {{t_x}, {t_y}} \right) = \left( {0, 0} \right), \left( {12, 0} \right), \left( {0, 12} \right)}$, (−12, 0), and (0, −12), respectively. Figure 5b shows the superimposed electron beam patterns on the detector plane when the detilt coils are switched off. Since the exposure time of the CCD camera is longer than the horizontal scan time, the patterns with the five different beam tilt angles are superimposed. By turning the detilt coils on, Fig. 5b becomes 5c. The optical conditions of Figs. 5b and 3b are the same (the aberration-corrected region is about 26 mrad and the convergence angle is 10 mrad). By comparing Figs. 5b and 3a, the CL aperture positions under the beam tilted conditions are confirmed to reside within the aberration-corrected area. Using the tilt-scan system with appropriate tilt angles makes it possible to reduce the blur of the probe and acquire clear images of gold nanoparticles, as shown in Fig. 5a. Gold nanoparticles can also be observed clearly in the averaged image of the five tilted images, as shown in Fig. 5d, confirming the precise probe position control under the beam tilted conditions.

**Fig. 6.**
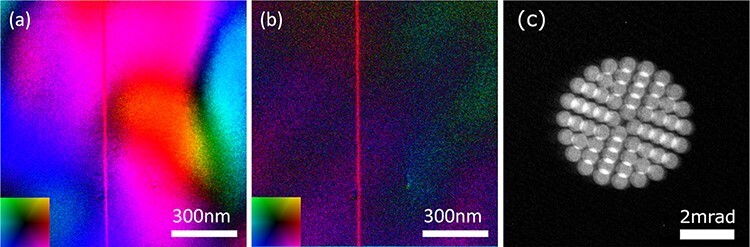
DPC STEM images of a p-n junction in GaAs. (a) Electron field vector color map of the p-n junction without the beam tilt. The inset shows the field directions. The observing direction is parallel to the GaAs [110] direction. The strong diffraction contrast is overlapped with the true electric field contrast. (b) Electron field vector color map of the p-n junction acquired by averaging 60 DPC STEM images with different tilt angles at the same sample position in (a). The electric fields from the left-hand side n-doped region to the right-hand side p-doped region at the p-n junction can be observed. (c) The electron beam pattern on the detector plane imaged by the detilt coils off with the CCD camera. The 60 positions of the CL aperture image, one of which corresponds to 0.3 mrad, show the tilt angle distribution used for obtaining (b).List: DPC, differential phase contrast; STEM, scanning transmission electron microscopy; CCD, charge-coupled device.

Figures 6a and b show the electric field maps of a p-n junction of GaAs observed from the [110] zone axis with and without the beam-tilt averaging. The convergence angle is set to 0.3 mrad to obtain high S/N for imaging a weak electric field at the p-n junction [11]. The number of the tilt angles is 60, and they are imaged by a CCD camera with the detilt coils off, as shown in Fig. 6c. There should be a vertical line-shaped electric field of a p-n junction near the center of the images. However, the contrast of the p-n junction electric field cannot be clearly observed in Fig. 6a due to the strong diffraction contrast. On the other hand, the diffraction contrast is largely reduced in Fig. 6b with the 60 images averaging. The pure electric field component can now be observed. The present result demonstrates that the tilt-scan system effectively reduces the diffraction contrast and thus highlights the true electromagnetic field structures inside specimens.

## Conclusions

We have developed a new beam scan system that can acquire averaged DPC STEM images with different beam incident angles to the specimens. The beam is tilted by optically moving the CL aperture in this system. Therefore, it is possible to suppress the blurring of the averaged images if the CL aperture resides within the aberration-free region of the Ronchigram. Furthermore, by synchronizing the tilt angle change with the flyback time of the scan, it is possible to suppress the probe shift on the specimen plane during the change of the beam tilt angles. We demonstrate that the gold nanoparticles can be clearly observed in the five ADF STEM images’ averaged images with different beam incident angles. This indicates that we can suppress image degradation due to aberration and probe shift in the averaged STEM images. Finally, it is shown that the diffraction contrast can be effectively reduced in DPC images, and the electric field of a p-n junction can be observed using this system. For the quantitative interpretation of the tilt averaged images, it will be necessary to consider the sample shape, including thickness, crystal structure, irradiation conditions and tilt angle distribution. It is expected that the strategy to select optimum imaging conditions for quantitative image analysis will be established if we apply this method to various types of samples and analyze the tilt averaged images quantitatively in the future. Using DPC STEM, the system will be a powerful tool for quantitative electromagnetic field observations for crystalline samples.
